# Effects of Low-Frequency Repetitive Transcranial Magnetic Stimulation of the Bilateral Parietal Cortex in Patients With Tourette Syndrome

**DOI:** 10.3389/fneur.2021.602830

**Published:** 2021-02-12

**Authors:** Mengmeng Fu, Hua Wei, Xianghong Meng, Hai Chen, Baoxiang Shang, Fuyong Chen, Zhaoyang Huang, Ying Sun, Yuping Wang

**Affiliations:** ^1^Department of Neurology, Xuanwu Hospital, Capital Medical University, Beijing, China; ^2^Department of Neurosurgery, Shenzhen University General Hospital, Shenzhen University, Shenzhen, China; ^3^Beijing Key Laboratory of Neuromodulation, Beijing, China; ^4^Center of Epilepsy, Beijing Institute for Brain Disorders, Capital Medical University, Ministry of Science and Technology, Beijing, China

**Keywords:** Tourette syndrome, repetitive transcranial magnetic stimulation, parietal cortex, sensory, supplementary motor area

## Abstract

**Background:** Traditional medical treatments are not effective for some patients with Tourette syndrome (TS). According to the literature, repetitive transcranial magnetic stimulation (rTMS) may be effective for the treatment of TS; however, different targets show different results.

**Objective:** To assess the efficacy and safety of low-frequency rTMS in patients with TS, with the bilateral parietal cortex as the target.

**Methods:** Thirty patients with TS were divided into two groups: active and sham groups. The active group was subjected to 0.5-Hz rTMS at 90% of resting motor threshold (RMT) with 1,200 stimuli/day/side, whereas the sham group was subjected to 0.5-Hz rTMS at 10% of RMT with 1,200 stimuli/day/side with changes in the coil direction. Both groups were bilaterally stimulated over the parietal cortex (P3 and P4 electrode sites) for 10 consecutive days. The symptoms of tics and premonitory urges were evaluated using the Yale Global Tic Severity Scale (YGTSS), Modified Scoring Method for the Rush Video-based Tic Rating Scale (MRVBTS), and Premonitory Urge for Tics Scale (PUTS) scores at baseline, the end of the 10-day treatment, 1 week after treatment, and 1 month after treatment.

**Results:** At the end of the 10-day treatment, the YGTSS total, YGTSS motor tic, YGTSS phonic tic, MRVBTS, and PUTS scores in the active group significantly improved and improvements were maintained for at least 1 month.

**Conclusions:** Low-frequency bilateral rTMS of the parietal cortex can markedly alleviate motor tics, phonic tics, and premonitory urges in patients with TS.

## Introduction

Tourette syndrome (TS) manifests as a variety of motor tics and at least one phonic tic lasting for more than 1 year (1). In most children with TS, tic symptoms may improve during adolescence; however, if symptoms persist into adulthood, the tics may become severe or chronic (2). The prevalence of TS varies based on age; it is seen in ~1–2% of school children and 0.3–0.5% of adults (3, 4). Premonitory urges are more common in patients with TS who typically describe them as uncomfortable cognitive or physical sensations prior to tics and strong motor urges (5–7). They may be internally generated and may prompt the release of motor or phonic tics as well as be associated with the severity of tics (8, 9).

Transcranial magnetic stimulation (TMS) is a non-invasive technology that was developed in 1985 (10). Repeated TMS (rTMS) has been proposed as a potential treatment for neurological diseases. Studies have shown that rTMS can modulate underlying cortical excitability and that the effect of rTMS is influenced by stimulation frequency, intensity, and stimulation pulse number and duration (11). High-frequency (>5 Hz) stimulation increases cortical excitability, whereas low-frequency (≤1 Hz) stimulation inhibits it (12). Therefore, this method may become a new and effective treatment approach for a variety of neuropsychiatric disorders (13–17). The efficacy of rTMS for the treatment of TS has been studied over the past decade (18–22).

The pathological processes of tics remain unclear. The combination of neuropathology and neuroimaging findings strongly supports the conclusion that there is a cortico-striatal-thalamo-cortical (CSTC) network dysfunction in patients with TS (6, 22, 23). According to their different functions, the CSTC loop is divided into different circuits, including sensorimotor, association, and limbic circuits, which are involved in movement, cognition, and motivated behavior (24). Several early studies used the left motor cortex or premotor cortex as targets to observe the role of 1-Hz rTMS in TS and found that the symptoms of TS tics were not significantly reduced (18, 19). Instead, studies on the treatment of TS with low-frequency rTMS targeting the supplementary motor area (SMA) have shown obvious therapeutic effects (12, 20, 21). This suggests that the effects of low-frequency rTMS for the treatment of TS depends on the target.

The involvement of the sensory part of the CSTC loop in TS has not been sufficiently valued. The parietal cortex can integrate different sensory information and influence the production of movement (25). In a functional magnetic resonance imaging (fMRI) study, Bohlhalter et al. (26) showed that the parietal lobe plays a role in tic generation. Two seconds before a tic, specific areas, including the parietal operculum and SMA, show prominent blood-oxygen-level-dependent (BOLD) activity. When a tic occurs, the areas with prominent activity include the bilateral superior parietal lobule (SPL). In another fMRI study, Neuner et al. (27) reported that the activation area includes the parietal cortex, SMA, and primary sensorimotor cortex 2 s before tic onset. They also stated that the putamen was activated 1 s before the tic and that the thalamus was activated when the tic occurred. Crucially, this study showed that cortical activity appears early in tic genesis; therefore, the role of the cortex should be emphasized (28). Davis et al. (29) reported that the parietal cortex variants significantly related to gene expression have a significant contribution toward the heritability of TS and OCD. Therefore, we hypothesized that the bilateral parietal cortex may be a new target for rTMS in patients with TS.

In the present study, we performed low-frequency rTMS in patients with TS for the following reasons. First, as mentioned earlier, before and during the onset of a tic, the parietal cortex is activated (26, 27). Second, studies have shown that patients with TS may have sensorimotor integration or gating abnormalities (30–33). Therefore, low -frequency rTMS acting on the parietal lobe may enhance the inhibitory effect. According to our previous study, rTMS showed significant efficacy in the treatment of patients with refractory partial epilepsy with 0.5-Hz rTMS at a 90% rest movement threshold (RMT) (34). Therefore, we hypothesized that bilateral stimulation of the parietal cortex at 0.5 Hz could improve the tic symptoms in patients with TS.

## Materials and Methods

### Subjects

Thirty subjects with TS from the Neurology Clinic of Xuanwu Hospital were included. The subjects met the diagnosis criteria of TS stated in the fourth edition of the Diagnostic and Statistical Manual of Mental Disorders (DSM-IV) (35) and had moderate-to-severe tic symptoms but without psychiatric comorbidities. All participants were 15–30 years old. The type and dose of medications remained stable for at least 2 months before trial registration. The exclusion criteria were as follows: patients with secondary TS, those with evidence or prior history of neurological or other physical diseases, pregnant women, those with substance or alcohol abuse or dependence, those with severe respiratory or cardiac diseases, and those with implantation of metal devices in their body. The 30 patients with TS were divided into two groups based on the matching method (*n* = 15 each): active rTMS and sham groups.

All patients or their guardians provided informed consent. This study was approved by the Ethics Committee of Xuanwu Hospital.

### Assessments

All assessments were performed by trained raters who were blinded to the study and did not know whether the patients belonged to the active or sham groups. All assessments were completed by the same raters. All patients were assessed using the Yale Global Tic Severity Scale (YGTSS) (36), Modified Scoring Method for the Rush Video-based Tic Rating Scale (MRVBTS) (37), and Premonitory Urge for Tics Scale (PUTS) (38). YGTSS was used to rate motor tics and phonic tics, with 0–25 points for each item. A separate rating for impairment is also included in YGTSS. The sum of the motor tic scores, phonic tic scores, and impairment ratings is equal to the YGTSS total score. In this study, for YGTSS, total scores and motor tic and phonic tic subscores were calculated. MRVBTS includes the number of body areas, frequency of motor tics, frequency of phonic tics, severity of motor tics, and severity of phonic tics, with a total score of 20 points. PUTS is a measure of the severity of premonitory urges in patients with TS.

The above scales were scored at baseline, the end of the 10-day treatment, 1 week after treatment, and 1 month after treatment.

### Treatment

The patients' RMT values were measured prior to treatment. The patients were made to sit in a comfortable chair in a relaxed manner and were asked not to suppress tics. Magstim (Magstim Ltd., UK) and a figure-of-eight coil were used. The bilateral RMT of the abductor pollicis brevis was measured. The lowest intensity that produced five motor-evoked potentials (≥50 μV) in 10 trials is defined as RMT (17).

During the treatment, both the active and sham groups used Magstim (Magstim Ltd, UK) and a figure-of-eight coil. In the active group, the frequency was 0.5 Hz, the intensity was 90% of RMT (independent of hemisphere), and the targets were the P3 and P4 electrode sites. Stimulation comprised three trains of 400 pulses per side (1,200 pulses/side/day) for 10 consecutive days. First, the figure-of-eight coil was used to stimulate three trains (1,200 pulses) at P3 and then used to stimulate three trains at P4 (1,200 pulses). The inter-train interval was 10 min. The cortical regions underlying the P3 and P4 electrode sites in the international 10–20 EEG system include the Brodmann area (BA) 40, BA 7, and BA 39 (39).

In the sham group, the frequency was also 0.5 Hz, the targets were the P3 and P4 electrode sites, and stimulation comprised three trains of 400 pulses per side (1,200 pulses/side/d) for 10 consecutive days. However, the intensity in the sham group was 10% of RMT (independent of hemisphere) (34). The stimulation coil was parallel to the tangent of the skull target in the active group, and the direction of the stimulation coil was at a 45° angle from that in the sham group, almost parallel to the ground.

### Statistical Analysis

Statistical analysis was performed using SPSS version 16.0 (SPSS Inc., USA). The statisticians were not aware of the grouping of the patients. The continuous variable *t*-test and categorical variable Fisher's exact test were used to compare demographic and clinical characteristics of the groups at baseline. The Kolmogorov–Smirnov test of normality was used for the scores of each scale. The group- and time-dependent effects of rTMS on YGTSS total scores, YGTSS motor tic scores, YGTSS phonic tic scores, MRVBTS scores, and PUTS scores were evaluated via repeated-measures analysis of variance (ANOVA) with adjustments for non-sphericity. The Greenhouse–Geisser correction results were used whenever necessary. A *P* < 0.05 was considered statistically significant.

## Results

All 30 patients completed the entire experiment without dropping out or missing the follow-ups. The demographic and clinical characteristics, including age, sex, course of disease, and baseline scale scores of all patients, are summarized in Table 1. There were no statistical differences in these characteristics between the two groups. All patients continued their usual medications at the same doses for ≥2 months before the trial; the medications included tiapride (dose ≤ 300 mg/day), sulpiride (dose ≤ 300 mg/day), haloperidol (dose ≤ 3 mg/day), and risperidone (dose ≤ 2 mg/day). Each patient either took no or only one medication.

**Table 1:** Demographic and clinical characteristics of the patients.

**Variable**	**Active group (*n* = 15)**	**Sham group (*n* = 15)**	***p*-value**
Age	19.73 (3.693)	19.80 (4.427)	0.965^a^
Gender (F/M)	12/3	12/3	1^b^
Disease duration (Y)	10.27 (4.636)	10.20 (4.329)	0.868^a^
YGTSS total	55.47 (8.601)	56.40 (7.567)	0.755^a^
YGTSS motor tics	17.00 (2.828)	17.47 (2.295)	0.624^a^
YGTSS phonic tics	13.13 (3.603)	13.60 (3.481)	0.721^a^
MRVBTS	12.33 (2.160)	12.40 (1.957)	0.930^a^
PUTS	21.13 (6.749)	22.53 (6.346)	0.563^a^

*Data are presented as mean (SD). YGTSS, Yale Global Tic Severity Scale; MRVBTS, Modified Scoring Method for the Rush video-based Tic Rating Scale; PUTS, Premonitory Urge for Tics Scale*.

a *The p-value was obtained by a two-sample two-tailed t-test*.

b *The p-value was obtained using a Fisher's exact test*.

**Figure 1:**
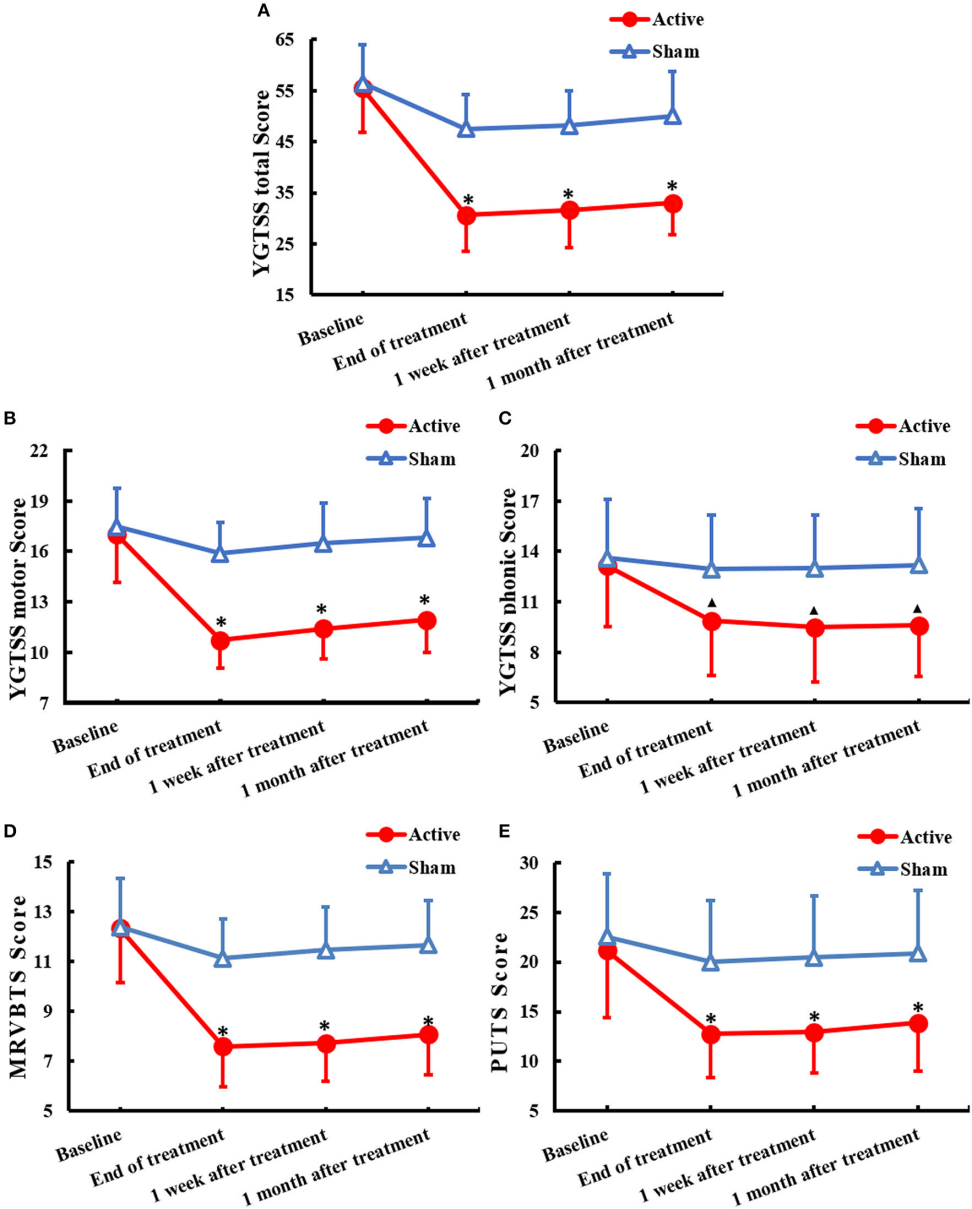
Changes of clinical rating scales(mean ± SD) of TS across 1 month after treatment of rTMS to the bilateral parietal cortex: **(A)** YGTSS total score, **(B)** YGTSS motor score, **(C)** YGTSS phonic score, **(D)** MRVBTS score, and **(E)** PUTS score. YGTSS, Yale Global Tic Severity Scale; MRVBTS, Modified Scoring Method for the Rush video-based Tic Rating Scale; PUTS, Premonitory Urge for Tics Scale. ^▴^*P* < 0.05 and *P < 0.01 between groups.

In the active group, tic symptoms, including winking, head shaking, throat clearing, shrugging, and premonitory urges, were significantly attenuated following active rTMS. However, in the sham group, tic symptoms and premonitory urges showed little changes. The scores of each scale were in accordance with the normal distribution. There was a significant decrease in the YGTSS total scale scores (*F* = 192.555, df = 1.828, *P* < 0.001) as well as a significant difference between the active rTMS and sham group (*F* = 26.282, *P* < 0.001); the improvement continued from the end of treatment to 1 month after treatment (*F* = 48.091, time × group interaction *P* < 0.001) (Figure 1A). Further, there was a significant decrease in the YGTSS motor tic scores (*F* = 141.363, df = 1.561, *P* < 0.001) as well as a significant difference between the active rTMS and sham groups (*F* = 26.661, *P* < 0.001), with a marked improvement from the end of treatment to 1 month after treatment (*F* = 90.501, time × group interaction *P* < 0.001) (Figure 1B). Moreover, there was a significant decrease in YGTSS phonic tic scale scores (*F* = 124.809, df = 2.266, *P* < 0.001) as well as a significant difference between the active rTMS and sham groups (*F* = 4.990, *p* = 0.034); the improvement continued from the end of treatment to 1 month after treatment (*F* = 66.966, time × group interaction *P* < 0.001) (Figure 1C). There was also a significant decrease in MRVBTS scores (*F* = 220.880, df = 1.691, *P* < 0.001) as well as a significant difference between the active rTMS and sham group (*F* = 19.188, *P* < 0.001), with a marked improvement from the end of treatment to 1 month after treatment (*F* = 90.157, time × group interaction *P* < 0.001) (Figure 1D). Lastly, there was a significant decrease in PUTS scale scores (*F* = 97.604, df = 1.503, *P* < 0.001) and a significant difference between the active rTMS group and sham group (*F* = 8.136, *p* = 0.008); the improvement continued from the end of treatment to 1 month after treatment (*F* = 32.587, time × group interaction *P* < 0.001) (Figure 1E).

The results suggest that for all patients in this study, rTMS treatment was safe and well-tolerated. During the study, no patient reported any signs of headaches, seizures, memory, attention impairments, or other side effects.

## Discussion

Our study demonstrated significant improvements in YGTSS motor tic, YGTSS phonic tic, YGTSS total, MRVBTS, and PUTS scores after parietal stimulation. The results of our study suggest that bilateral rTMS of the parietal lobe at 0.5 Hz and 90% RMT could significantly improve motor tics, phonic tics, and premonitory urges without complications. The effect was significant and was observed after 10 days of treatment and lasted for at least 1 month.

As mentioned earlier, the effectiveness of rTMS treatment highly depends on the stimulation target. The bilateral parietal lobe is an effective target for rTMS stimulation. However, the underlying mechanism of bilateral rTMS of the parietal lobe used to treat patients with TS remains unclear. The parietal lobe plays a role in the generation of tics and requires targeted treatment. It is part of the association cortex that can integrate various sensations, select the most appropriate movement, and participate in final precise execution (40). Therefore, the parietal cortex is closely related to movement. In the parietal lobe, motor intention may be related to sensory integration (41). A study showed that in humans, when the posterior parietal cortex was stimulated, there was a strong desire to move (42). In humans, including patients with TS, the parietal lobe is involved in motor planning and decision-making and plays an important role in these processes. The functional areas of motor planning, preparation and execution, and somatosensory perception are related to the generation and expression of tics in patients with TS (28, 42, 43). Therefore, bilateral low frequency rTMS of the parietal lobe in TS might disrupt the preparation and execution of tics.

Previous studies have suggested that the function and structure of the parietal lobe are abnormal in patients with TS. Bohlhalter et al. (26) and Neuner et al. (27) found that abnormal activation of the parietal lobe played a role in the generation, preparation, and execution of tics. Wang et al. (44) found that while releasing spontaneous tics, the primary somatosensory and posterior parietal cortexes had strong activity and interregional causality. In addition, the regions with significant correlations with current tic severity included the posterior parietal cortex. The severity of tic symptoms may be modulated by changes in the neuroplasticity of associated circuits (45). Several studies have shown that patients with TS have multifocal cortical thinning (46–50). Fahim et al. (47) revealed that the right parietal lobe of patients with TS thins with age. However, the severity of patients with TS negatively correlated with the average thickness of the somatosensory–motor and parietal–orbitofrontal cortex. Peterson et al. (50) reported that symptom severity significantly and negatively correlated with parietal lobe and orbitofrontal volumes. This finding suggests that this portion of the action-attention system has small volumes and may have an insufficient inhibitory reserve to help suppress these unwanted behaviors. The number of GABAergic neurons in the cortex may be one of the reasons for cortical thinning in patients with TS (46). Therefore, the structure and function of the parietal cortex are abnormal in patients with TS. In our study, low-frequency bilateral rTMS targeting the parietal cortex might modulate the function of the parietal cortex and enhance its suppression.

In this study, low-frequency bilateral rTMS of the parietal lobe improved both motor and phonic tics. Stern et al. (51) performed [^15^O]H_2_O-positron emission tomography in patients with TS and found that for different types of tics, the activated cortex and subcortical regions were different, as were the clinical manifestations. Coprolalia was not only associated with activation in the region of the Broca's area and frontal operculum but also with the other language regions, including the posterior superior temporal gyrus and supramarginal gyrus. Motor tics were associated with activation in a region deep within the inferior parietal, sensorimotor cortex, superior temporal gyrus, and somatosensory cortex. Therefore, both motor tics and phonic tics are related to parietal lobe activity. This may be the reason for the improvements in both motor and phonic tics in this study.

Although the main clinical manifestations of TS are motor tics and phonic tics, sensation plays an important role in the pathophysiology of TS. At present, the most studied sensory symptom is premonitory urges. Studies have confirmed that premonitory urges are associated with the occurrence and severity of tics. Using fMRI, Wang et al. (44) discovered that in patients with TS, the activities produced by spontaneous tics in the posterior parietal cortex and somatosensory cortex were stronger than those produced by voluntary tics. They suggested that the activities in these regions might represent the characteristics of the premonitory urges that generate spontaneous tics. As mentioned earlier, patients with TS have sensorimotor integration deficits, which are thought to be associated with premonitory urges (52). The above studies might explain why low-frequency bilateral rTMS of the parietal cortex in TS improves premonitory urges.

There were possible confounding effects of medications in our study. To minimize the confounding effects of medications, all patients remained on their usual medications at the same doses for at least 2 months. Each patient either took no medication or only one medication; the type of medicine taken was small and the dose was low. Nevertheless, we still cannot completely rule out the influence of medications because these medications may alter brain excitability and therefore have an effect on rTMS (53).

In summary, with bilateral stimulation of the parietal lobe, 0.5-Hz rTMS is effective in patients with TS. The potential mechanism may involve the regulation of the parietal cortex activity, enhancement of parietal lobe suppression, reduction of sensory system activity, reduction of sensory motor cortex output, and disruption of tic preparation and execution.

This study has some limitations. This study did not explore the effective mechanism of bilateral rTMS of the bilateral parietal cortex at 0.5 Hz for the treatment of TS. There are no neurophysiological measures; therefore, future research could use fMRI and electrophysiology to detect changes in the activity and excitability of the cortical and CSTC loops generated by rTMS. In addition, the bilateral parietal cortex could not be located using a neuronavigation system. There are individual differences in the positioning of the international EEG 10–20 system. It cannot be ruled out that the difference in the efficacy of patients with TS is related to differences in the precise location of the stimulation. Moreover, future studies should use a control with other stimulation sites to better illustrate the role of the parietal lobe as a stimulation target. The sample size of this study was small; therefore, future studies with larger samples are warranted to confirm our conclusions.

## Conclusion

Our research suggested that with the left and right parietal cortex as target sites, 0.5-Hz rTMS was effective and safe in the treatment of TS patients and significantly improved motor tics, phonic tics, and premonitory urges. The parietal lobe could be a new and effective target for rTMS in the treatment of TS. The mechanisms underlying the therapeutic effect may involve regulation of parietal cortex activity, enhancement of parietal lobe suppression, reduction of sensory system activity, reduction of sensory motor cortex output, and disruption of tics preparation and execution.

## Data Availability Statement

The raw data supporting the conclusions of this article will be made available by the authors, without undue reservation.

## Ethics Statement

The studies involving human participants were reviewed and approved by Xuanwu Hospital Ethics Committee, Xuanwu Hospital, Capital Medical University. Written informed consent to participate in this study was provided by the participants' legal guardian/next of kin.

## Author Contributions

MF, YS, and YW contributed at all stages of manuscript preparation. MF wrote the manuscript. All authors contributed to manuscript revision, read, and approved the submitted version.

## Conflict of Interest

The authors declare that the research was conducted in the absence of any commercial or financial relationships that could be construed as a potential conflict of interest.
